# Expression of AdipoR1 and AdipoR2 Receptors as Leptin-Breast Cancer Regulation Mechanisms

**DOI:** 10.1155/2017/4862016

**Published:** 2017-09-05

**Authors:** Martha Daniela Mociño-Rodríguez, Jonnathan Guadalupe Santillán-Benítez, David Salomón Dozal-Domínguez, María Dolores Hernández-Navarro, Miriam Veronica Flores-Merino, Antonio Sandoval-Cabrera, Francisco Javier García Vázquez

**Affiliations:** ^1^Faculty of Chemistry, Autonomous University of the State of Mexico (UAEMex), Toluca, MEX, Mexico; ^2^Specialized Hemato-Oncological Laboratory of the Children's Hospital, IMIEM, Toluca, MEX, Mexico; ^3^Molecular Biology Laboratory, Medical Research Center (CICMED), Toluca, MEX, Mexico; ^4^Laboratory of Immunohistochemistry, National Pediatrics Institute, Coyoacán, MEX, Mexico

## Abstract

The development of breast cancer is influenced by the adipose tissue through the proteins leptin and adiponectin. However, there is little research concerning AdipoR1 and AdipoR2 receptors and the influence of leptin over them. The objective of this work was to analyze the expression of AdipoR1 and AdipoR2, modulated by differential concentrations of leptin in an obesity model (10 ng/mL, 100 ng/mL, and 1000 ng/mL) associated with breast cancer in MCF-7 and HCC1937 cell lines. Each cell line was characterized through immunohistochemistry, and the expression of AdipoR1 and AdipoR2 was analyzed by PCR in real time using TaqMan® probes. Leptin induced an increase in cell population of MCF-7 (23.8%, 10 ng/mL, 48 h) and HCC1937 (17.24%, 1000 ng/mL, 72 h). In MCF-7, the expression of AdipoR1 decreased (3.81%, 1000 ng/mL) and the expression of AdipoR2 increased by 13.74 times (10 ng/mL) with regard to the control. In HCC1937, the expression of AdipoR1 decreased by 86.28% (10 ng/mL), as well as the expression of AdipoR2 (50.3%, 100 ng/mL). In regard to the results obtained, it could be concluded that leptin has an effect over the expression of AdipoR1 and AdipoR2 mRNA.

## 1. Introduction

Over the last 20 years, the development of breast cancer has been associated to lifestyle changes, primarily to obesity [1]. Obesity is considered an excess in the accumulation of adipose tissue produced when caloric intake exceeds energy consumption [2]. Besides storing excess calories in the form of lipids, the adipose tissue participates through diverse forms in the development of cancer due to the fact that it also acts as an endocrine gland, liberating the adipocytokines leptin and adiponectin and proinflammatory molecules [3].

Leptin is a protein produced from the OB gen or “gen of obesity” [4]. The expression of the gen is restricted to adipose tissue, and its levels are closely related to the storage of triglycerides and the mass of adipose tissue [5]. It is a pleiotropic adipocytokine which regulates the ingestion of foods, energy consumption, immunity, inflammation, hematopoiesis, cell differentiation, and proliferation [6, 7].

Adiponectin is a protein produced exclusively in white adipose tissue [8] and is considered as a protection hormone, carrying out an important role in the regulation of glucose through a potent sensitizing effect towards insulin, which affects the uptake of glucose in the muscle and participates in the homeostasis of lipids [9]. Its plasmatic concentration has an inversely proportional relationship towards obesity, body mass index, accumulation of visceral fat, and insulin resistance [10, 11]. It carries out its function through two specific receptors, AdipoR1 and AdipoR2, which are receptors coupled to G proteins with six transmembrane domains. AdipoR1 is predominantly expressed in striated muscle, while AdipoR2 is mainly expressed in the liver [12]. The bonding of adiponectin to its receptors increases the activity of the AMP-dependent protein kinase (AMPK) and the peroxisome proliferator-activated alpha receptor (PPAR-*α*), favoring the oxidation of fatty acids and the entrance of glucose into tissues [13]. The activated AMPK system plays a vital role in the regulation of the energetic metabolism and cell quiescence [14, 15].

The pathologies, which modify the biology of adipose tissue, such as obesity, and produce an alteration in the secretion of these adipocytokines, increasing the liberation of leptin, VEGF, IL-6, and TNF-*α* and decreasing the secretion of adiponectin could be linked to different carcinogenic mechanisms including cell differentiation, apoptosis, cell proliferation, angiogenesis, and alteration of steroidal sex hormone levels [9].

This variation in the secretion of adipocytokines by the adipose deposits may explain the relationship between obesity and the development of breast cancer, since obese subjects present a decrease in the levels of circulating adiponectin and increase in the plasmatic concentration of leptin [5, 16].

The aforementioned has been observed in diverse *in vitro* experiments, in which the promoting activity of proliferation in breast cancer cell lines by leptin is mediated through different signal pathways; on one hand, it acts by inducing the P13K/Akt survival pathway, activating the phosphorylation of Akt, stimulating protein expression of PKC-alpha, activating the MAPK pathway, and stimulating ERK1 and ERK2 [17–20]. It activates the STAT3 pathway and induces upregulation of c-myc at an mRNA level, as well as at a protein level. Likewise, it upregulates genes of the cell cycle (cyclin D1) and reduces the expression of p53 and the production of Bax [21–23]. Some studies suggest that leptin may promote angiogenesis in breast cancer through the signaling of VEGF [24]. In this manner, it can be deduced that the procarcinogenic effect of leptin derives from the activation of signaling pathways implicated in the cell proliferation process and a downregulation of the apoptotic response [25, 26].

Likewise, the antiproliferative action of breast cancer cells by adiponectin is mediated by an inactivation of protein 1 and 3 expression in MAPK p44/42, a stimulation of AMPK activity and a decrease in phosphorylation of Akt, associated with an increased expression of LKB1, reducing the activity of mTOR and leading to a decrease in the production of reactive oxygen species [19, 27–29]. Studies suggest that adiponectin modulates the cell cycle of breast cancer cells, decreasing the expression of cyclin D1 [30, 31]. In diverse studies, it has been detected that adiponectin induces cell apoptosis when a stimulus is given to cell lines with an incubation period longer than 48 h [32, 33]. Also, an increase of PPAR in estrogen-positive cell lines, the downregulation of Bcl2, and the activation of p53 and Bax have been detected [34, 35]. Finally, it is suggested that an interaction of adiponectin and estrogen pathways exists, since it is capable of reducing aromatase and the expression of the receptor [36].

For the identification of the procarcinogenic mechanisms of leptin, the breast cancer cell lines MCF-7, MDA-MB-231, and T47D have been characterized for the protein and its receptor [Ob-R), studying its effect over cell growth in the MCF-7, SK-BR-3, T47D, and ZR-75-1 cell lines. An increase in cell proliferation when the cells receive a single stimulus of leptin and incubated between 24 and 96 h has been observed [21, 27, 28, 37, 38].

In order to better understand the potential involvement of leptin in the expression of adiponectin receptors, AdipoR1 and AdipoR2, in the cells of breast cancer, the main objective of this research was to demonstrate that AdipoR1 and AdipoR2 were expressed in MCF-7 and HCC1937 breast cancer cell lines, as well as to determine and analyze the expression of these receptors modulated by differential concentrations of leptin [27].

## 2. Materials and Methods

### 2.1. Cell Culture

For this study, cell lines MCF-7 and HCC1937 were employed and were obtained from the American Type Culture Collection (ATCC). They were cultivated at 37°C in an atmosphere of 5% CO2 and 85% humidity, with culture medium (RPMI 1640 medium, Gibco®, Thermo Fisher Scientific™, Dulbecco's modified Eagle's medium, Caisson Labs, USA) supplemented with 10% fetal bovine serum (Gibco, Thermo Fisher Scientific, USA), inactivated by heat (30 min at 57°C; water bath), with medium exchange every third day.

### 2.2. Immunohistochemistry

The expression of estrogens (RE), progesterone (RP), Her2, and Ki67 was determined through immunohistochemistry. Once a confluence of 70% of cells was reached, they were washed with PBS (phosphate-buffered saline, Gibco, Thermo Fisher Scientific, USA) and were suspended with 0.05% trypsin (Gibco, Canada). The recuperated cells were centrifuged for 3 min at 1500 rpm, and the cell pellet was then used to prepare slides fixed with acetone. Posteriorly, the recuperation of epitopes was done, utilizing 0.1% sodium citrate (Dako, USA, pH 6.2), inactivating endogenous peroxidase with 0.9% hydrogen peroxide. The slides were washed with distilled water and were placed in PBS for 5 minutes [39]. The cells were then incubated during 45 minutes with the following antibodies: RE 1 : 35 (Dako, USA), RP 1 : 50 (Dako, USA), Her2 1 : 50 (Biocare, USA), and Ki67 1 : 100 (Biocare, USA).

Next, a two-step detection system was incorporated (Mach 1 Universal HRP-Polymer Detection, Biocare, USA) during 30 minutes each. The reaction was visualized with 3,3′-diaminobenzidine (Leica Biosystems, USA) and was stained with Hill's hematoxylin [39].

### 2.3. Proliferation Assay

The study of cell proliferation was carried out through the crystal-violet assay. For this purpose, two groups of 5000 MCF-7 and HCC1937 cells were inoculated in 96-well culture plates and were left to grow during 24 h (group 1) and 48 h (group 2). Afterwards, each group was washed every 24 h, during 96 h, with PBS and fresh culture medium (supplemented with 5% SFB) with leptin stimuli, which was added (Recom Hu Leptin Active, Sino Biological, Thermo Fisher Scientific, USA) in concentrations of 0 ng/mL (negative control), 10 ng/mL, 100 ng/mL, and 1000 ng/mL [27]. The decrease of SFB was done to reduce the growth factors that could interfere with the action of leptin [40].

Cell proliferation in each cell line was determined in triplicate for every concentration evaluated. After 24 h of stimulation, the cells were fixed with 1.1% glutaraldehyde (Merck, Germany) during 15 minutes and then PBS was added until the 96 h of stimulation with leptin was concluded for each group. At the end of stimulation, the fixed cells were stained with crystal-violet for 15 minutes, the plate was washed with water, and 10% acetic acid was placed in each well. Immediately after, the plate was read at an excitation wavelength of 490 nm and an emission wavelength of 630 nm in a microplate reader (Stat Fax 2100 Microplate Reader, Awareness Technology Inc.)

### 2.4. Analysis of Microassays

The regulation of gene expression of adiponectin receptors AdipoR1 and AdipoR2 was evaluated by means of the addition of leptin to the cell lines following the methodology proposed by Jardé et al., [27], with slight modifications. 2000 MCF-7 and HCC1937 cells were cultured in 24-well culture plates, allowing them to grow during 48 h.

After 24 h, 48 h, 72 h, and 96 h, the cells were washed with PBS and fresh culture medium (supplemented with 5% SFB) with leptin stimuli (0 ng/mL, 10 ng/mL, 100 ng/mL, and 1000 ng/mL) was added. At the end of the stimulation, the culture medium was removed and 400 *μ*L of TRIzol® reagent was added to carry out the mRNA extraction, in accordance with the protocol of use (Ambion™).

Once the mRNA was obtained, the acquisition of the complementary DNA was carried out (cDNA) from 2 *μ*g of mRNA through the reverse transcriptase reaction, in accordance with the protocol of use (high-capacity RNA to cDNA kit, Applied Biosystems), obtaining 20 *μ*L of cDNA.

The cDNA of the controls of each cell line were used to determine, through endpoint PCR (HotStarTaq DNA Polymerase, QIAGEN, Germany), the presence of AdipoR1 mRNA (Fw: 5′-AATTCCTGAGCGCTTCTTTCCT-3′, Rv: 5′-CATAGAAGTGGACAAAGGCTGC-3′) and AdipoR2 mRNA (Fw: 5′-TGCAGCCATTATAGTCTCCCAG-3′, Rv: 5′-GAATGATTCCACTCAGGCCTAG-3′), using the ACTB control gen (Fw: 5′-GCACCACACCTTCTACAAAG-3′, Rv: 5′-GGTCTCAAACATGATCTGGTC-3′).

Likewise, the cDNA obtained through the stimuli was used to determine the gene expression of AdipoR1 and AdipoR2 through real-time PCR (TaqMan Universal Mix II, with UNG), utilizing TaqMan probes (Gene Expression Assays, Applied Biosystems) for AdipoR1 (Hs 01114951_ml) and AdipoR2 (Hs 00226105_ml), using the ACTB gene as a control (Hs 99999903_ml), following the instructions of the LightCycler® 2.0 thermocycler (Roche Diagnostics). The program utilized to carry out the PCR was 50°C for 2 minutes, 95°C for 10 min, 40 cycles at 95°C for 15 seconds, and 60°C for 1 min.

For the determination of relative levels of AdipoR1 and AdipoR2 gene expression, the Pfaffl method of relative quantification, based on the comparison of the threshold cycle of a constitutive gene (mRNA of ACTB) with the test gene of each sample in duplicate, was carried out. The change in the fold of AdipoR1 and AdipoR2 was normalized with regard to the reference genes expressed constitutively and was then compared with the nontreated controls as follows: 2^−ΔΔCT^, where ΔΔCT = ΔΔCT = (*C*_T−target_ − *C*_T−reference_)_treated sample_ − (*C*_T−target_ − *C*_T−reference_)_calibrating sample_. “Calibrating sample” refers to the level of expression (1×) of the target gene normalized for the constitutive gen. The calibrator was chosen from the control group of each cell line and was given a value of 1 for relative expression [41–43].

## 3. Statistical Analysis

Statistical analysis was performed using GraphPad Prism 6 software. To analyze the results of the growth curves of each cell line, a two-factor ANOVA was performed with a *p* value > 0.05, to establish the relationship between incubation time and leptin concentrations.

For the analysis of gene expression results of AdipoR1 and AdipoR2, we first tested a normality hypothesis using the Kolmogorov-Smirnov test. Subsequently, the Student's *t*-test was performed for the parametric variables and the Mann–Whitney *U* test for the nonparametric variables with a *p* value > 0.05 for each one.

## 4. Results

### 4.1. Characterization by Immunohistochemistry

The selection of the cell lines employed in this experiment was done in accordance with the molecular classification of breast cancer, in other words, in accordance with the hormonal receptors that are found expressed by the cells.

**Figure 1:**
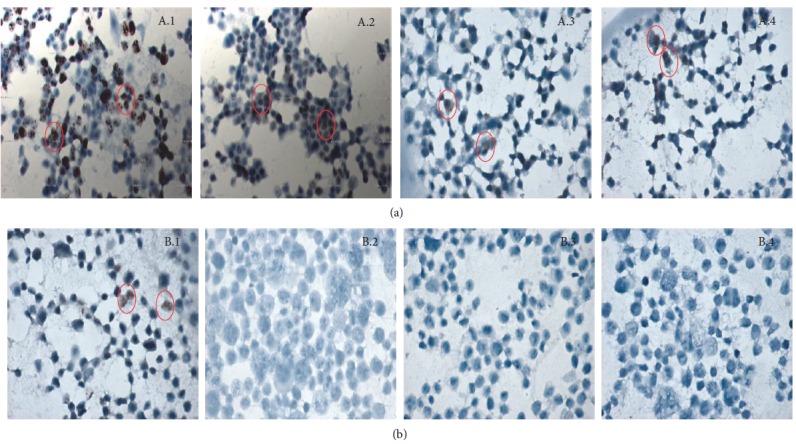
Characterization through immunohistochemistry of MCF-7 and HCC1937 cells. Detection through immunohistochemistry of the Ki67 protein, estrogen receptor (ER), progesterone receptor (PR), and Her2 in breast cancer cell lines. MCF-7 expression positive for Ki67 (A.1), ER (A.2), PR (A.3), and Her2 (A.4). HCC1937 cells exhibited an expression positive for Ki67 (B.1), while they showed an expression negative for ER (B.2), PR (B.3), and Her2 (B.4).

Using the immunohistochemistry technique, each cell line was characterized according to the molecular classification “triple positive” (MCF-7) and “triple negative” (HCC1937), evaluating the presence of Ki67, estrogen (RE), progesterone (RP), and Her2 (Figure 1).

MCF-7, a cell line identified as “triple positive,” had an expression positive for Ki67 at 78% (78 cells out of 100), RE at 45% (45 cells out of 100), RP at 5% (5 cells out of 100), and Her2 at 14% (14 cells out of 100).

In contrast to MCF-7, HCC1937 breast cancer cells presented an expression negative for RE, RP, and Her2. For Ki67, they presented an expression positive at 7% (7 cells out of 100), as shown in Figure 1.

### 4.2. Expression of Leptin, Adiponectin, AdipoR1, and AdipoR2 in Breast Cancer Cell Lines

**Figure 2:**
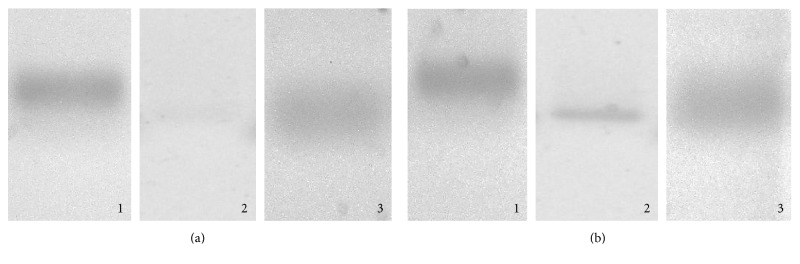
Detection of the expression of ABL control gene mRNA (1), adiponectin (2), and leptin (3) through endpoint PCR in MCF-7 (a) and HCC1937 (b) cells.

The mRNA of leptin and adiponectin was detected in MCF-7 breast cancer cells through endpoint PCR, observing a greater expression of leptin mRNA when compared to adiponectin mRNA, in accordance with the bands obtained in the electrophoretic gel, as shown in Figure 2. With regard to HCC1937, a triple-negative cell line with a mutation in BRCA1 [44], it was also possible to determine the expression of these genes (Figure 2).

**Figure 3:**
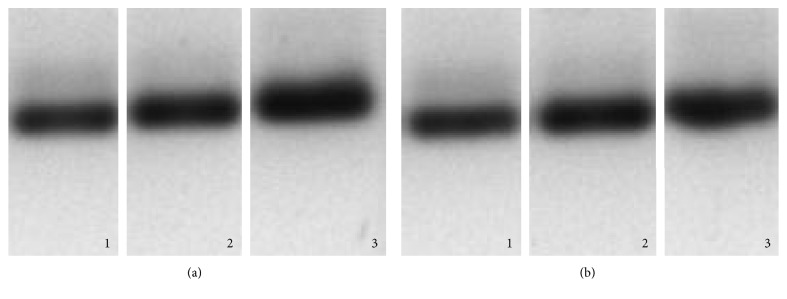
Detection of ACTB mRNA expression (1), AdipoR1 (2), and AdipoR2 (3) through endpoint PCR in MCF-7 (a) and HCC1937 cells (b).

As far as AdipoR1 and AdipoR2 mRNA is concerned, it was detected in MCF-7 and HCC1937 cells through endpoint PCR. In MCF-7, two adiponectin receptors, AdipoR1 and AdipoR2, were found to be expressed, just as Jardé et al. had reported [27]. As in the case of leptin and adiponectin, HCC1937 expresses these two receptors, a line that was not found to be reported “positive” in literature (Figure 3).

In both cell lines, the expression of AdipoR2 mRNA is greater with regard to BACT and AdipoR1 mRNA expression, in agreement with the bands obtained in the electrophoretic gel, as can be seen in Figure 3.

Once it was proven that the three cell lines studied express AdipoR1 and AdipoR2 mRNA, the quantitative determination of the expression of these receptors through real-time PCR, utilizing BACT as a control, went underway.

### 4.3. Effect of Leptin over Cell Proliferation of MCF-7 and HCC1937 Breast Cancer Cells

**Figure 4:**
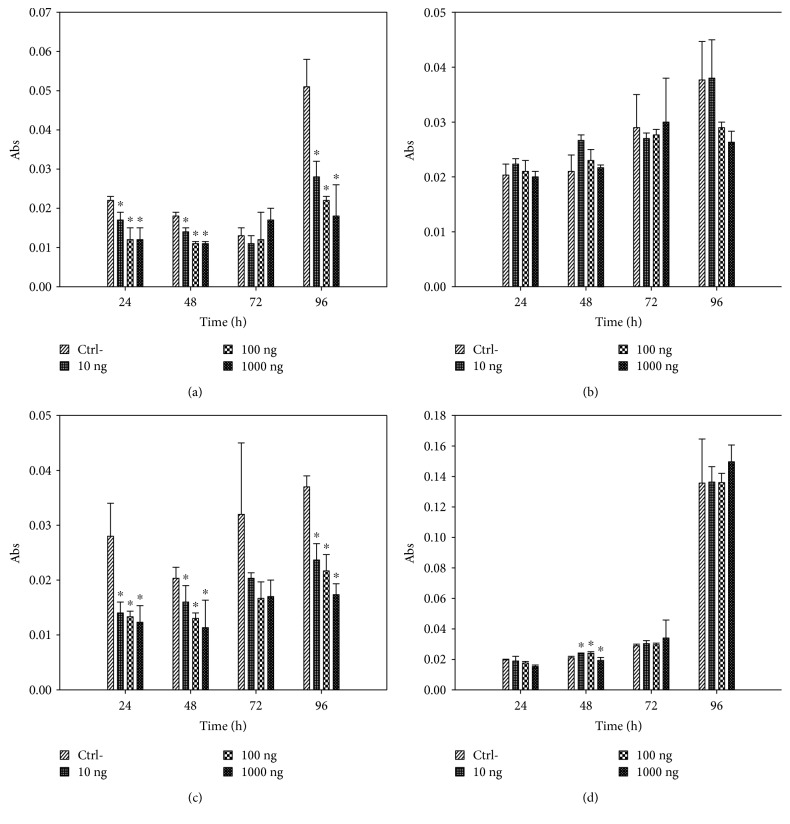
Cell viability with leptin stimulation. The data is expressed in mean ± SD, *n* = 3. Effect of leptin in MCF-7 breast cancer cells (a) at application of the first stimuli 24 h after cell culture, (b) MCF-7 cells at application of first stimuli 48 h after cell culture, (c) HCC1937 cells at application of first stimuli 24 h after cell culture, and (d) HCC1937 cells at application of first stimuli 48 hours after cell culture. ^∗^Significant differences compared with the control (ANOVA test, *p* < 0.05).

In the cells which were subjected to the leptin stimuli 24 h after culture (group 1), a negative effect was observed (antiproliferative) in cell proliferation in relation to leptin concentrations. With regard to MCF-7 cells, the cell population decreased 64.71% (1000 ng/mL, 96 h), except at 1000 ng/mL, where it increased 30.77% after 72 h of stimulation. The cell growth of HCC1937 decreased at all concentrations, up to 57.14% (1000 ng/mL, 24 h) (Figure 4).

In relation to the cells that were subjected to the leptin stimuli 48 h after culture (Group 2), a positive effect was observed in cell proliferation with relation to leptin concentrations. With regard to MCF-7, the effect of this protein over cell proliferation was positive, increasing by 28.57% (10 ng/mL, 48 h); this effect was maintained until 72 h, at a concentration of 1000 ng/mL, where there was a 3.45% increase with regard to the control. It was observed that for the concentration that corresponds to normal weight (10 ng/mL), the positive effect of leptin was maintained until 48 h and decreased by 6.90% with the third stimuli (72 h) and posteriorly presented an increase in the cell population once again at 96 h; not so for the concentration of 100 ng/mL, where at 72 h of stimulation, the cell population decreased by 26.68%. The cell growth of HCC1937 decreased by 23.46% (1000 ng/mL, 24 h), except after 72 h, at which all the concentrations presented an increase in the cell population, up to 17.24% (1000 ng/mL), maintaining themselves until 96 h, where there was an increase of 0.49% at 10 ng/mL, 0.25% at 100 ng/mL, and 10.31% at 1000 ng/mL with regard to the control, indicating that the higher the concentration of leptin, the higher the increase in cell population. It was observed that, despite the fact that the cell population was maintained above the control at all times and concentrations after 48 h of stimulation, there was a gradual decrease of cells, as can be seen in Figure 4.

### 4.4. Expression of AdipoR1 and AdipoR2 Mediated by Leptin in Breast Cancer Cell Lines

The mRNA of AdipoR1 and AdipoR2 was detected in MCF-7 and HCC1937 cell lines through real-time PCR. The expression of AdipoR1 and AdipoR2 in mRNA was determined in cells that were stimulated with leptin at differential concentrations (0 ng/mL, 10 ng/mL, 100 ng/mL, and 1000 ng/mL) in both cell lines.

**Figure 5:**
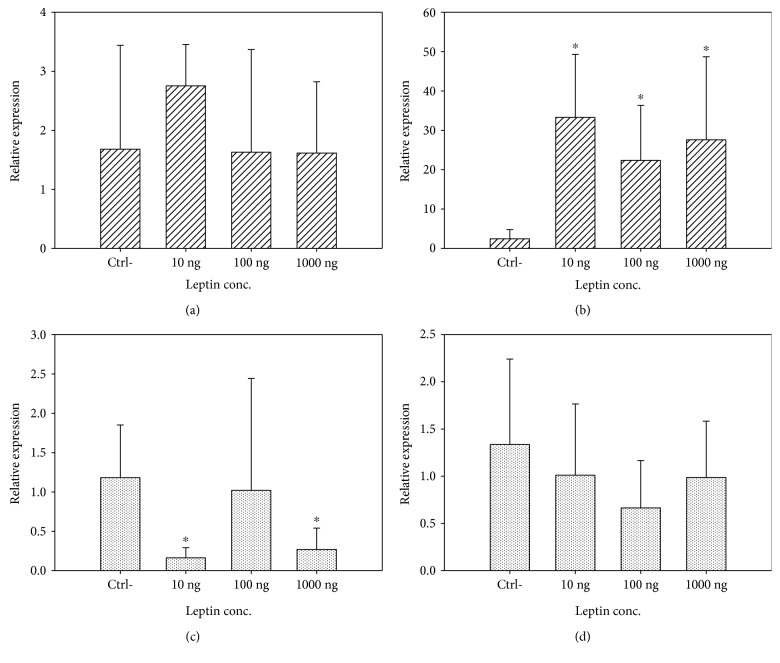
Units of relative expression of AdipoR1 and AdipoR2. The data is expressed as mean ± SD. In MCF-7 breast cancer cells, there is a 2.92% decrease in the expression of AdipoR1 (a) in cells treated with leptin (100 ng/mL) with regard to the control and of 3.81% for the concentration of 1000 ng/mL, except at 10 ng/mL, where there was a 64.03% increase; the expression of AdipoR2 (b) increased by 13.74 times in cells treated with leptin (10 ng/mL) with regard to the control, 9.23 times for 100 ng/mL, and 11.39 times for 1000 ng/mL, finding significant differences compared to the control in all concentrations. With regard to the HCC1937 breast cancer cells, the expression of AdipoR1 (c) presented an 86.28% decrease in cells treated with leptin (10 ng/mL) with regard to the control, 13.72% (100 ng/mL), and 77.14% (1000 ng/mL), finding significant differences for 10 g/mL and 1000 ng/mL; the expression of AdipoR2 (c) exhibited a 24.4% decrease in cells treated with leptin (10 ng/mL), with regard to the control, 50.3% (100 ng/mL), and 26.2% (1000 ng/mL); however, no significant differences were found in any of the concentrations relative to control. ^∗^Significant differences compared to the control (*U* of Mann–Whitney, *p* ≤ 0.05).

In MCF-7, the expression of AdipoR1 had a decrease of 3.81% (1000 ng/mL) in the cells treated with leptin with regard to the control, except for 100 ng/mL, where there was a 64.03% increase in the expression of the gene. However, the expression of AdipoR2 increased considerably in the cells treated with leptin (up to 13.74 times; 10 ng/mL), with regard to the control (Figure 5).

Analyzing the normalized values of treated cells in the MCF-7 line, no statistically significant differences were found in the expression of AdipoR1 between the controls and the cells treated with differential concentrations of leptin: 10 ng/mL (*p* = 0.0945), 100 ng/mL (*p* = 0.760), and 1000 ng/mL (*p* = 0.7840). However, upon analyzing the normalized values of treated cells for the expression of AdipoR2, statistically significant differences were found between the controls and the cells treated with differential concentrations of leptin: 10 ng/mL (*p* = 0.0022), 100 ng/mL (*p* = 0.0022), and 1000 ng/mL (*p* = 0.0022) (Table 1).

In HCC1937, the expression of AdipoR1 decreased in cells treated with leptin with regard to the control, as much as 86.28% (10 ng/mL), while the expression of AdipoR2 had a decrease in the cells treated with leptin with regard to the control, as much as 50.3% (100 ng/mL), which can be seen in Figure 5.

Analyzing the normalized values of treated cells in the HCC1937 line, statistically significant differences were found with regard to the controls for the expression of AdipoR1 in the cells treated: 10 ng/mL (*p* = 0.0043) and 1000 ng/mL (*p* = 0.0130). For the cells treated with 100 ng/mL of leptin, no statistically significant differences were found for the expression of AdipoR1. As for the expression of AdipoR2, upon analyzing the normalized values of treated cells, no statistically significant differences were found between the controls and the cells treated with differential concentrations of leptin: 10 ng/mL, 100 ng/mL, and 1000 ng/mL (Table 1).

## 5. Discussion

It has been determined that obesity and excess weight are risk factors for the development of breast cancer in women (occidental and Asian) [45, 46]. The procarcinogenic effect of leptin and the anticarcinogenic effect of adiponectin is mainly due to two mechanisms: (1) modulation of signaling pathways implicated in the proliferation process and (2) subtle regulation of the apoptotic response [4].

The characterization of each cell line through the immunohistochemistry technique was carried out according to the molecular classification “triple negative” (MCF-7) and “triple negative” (HCC1937), which allowed for the observation that in MCF-7, the estrogen receptor presented an expression 9 times greater than the progesterone receptor and up to 3.21 times greater than Her2. HCC1937 presented a negative expression for RF, PR, and Her2, which corresponds to that reported in the literature [47], in addition to the 7% Ki67 expression.

The effect of leptin over cell growth has been determined in MCF-7, MDA-MB231, SK-BR-3, T47D, and ZR-75-1 breast cancer cell lines, mediated by diverse signaling pathways when a single stimulus of the protein is applied and is incubated between 24 and 96 h [21, 27, 37, 38, 48, 49]. In this study, protein stimuli was applied every 24 h in order to emulate the constant secretion of the said protein by adipose tissue in a condition of normal weight (10 ng/mL), excess weight-obesity (100 ng/mL), and a very high concentration of leptin (1000 ng/mL).

In group 1 of the MCF-7 breast cancer cells, it was observed that the effect of leptin over cell proliferation was antiproliferative, decreasing up to 64.71%. It was also observed that this antiproliferative effect is due to the sensibility of the cells while being recultured (period of cellular adaptation) and to the immediate administration of an exogenous agent, since despite that there was a decrease in the cell population during the first stimulation with leptin (24 h), in the posterior stimulations (48 and 72 h), there was a gradual increase in cell population with regard to the population obtained at 24 h of stimulation. An increase of 0.65% and 9.50% for the concentration of 10 ng/mL at 48 h and 72 h, an increase of 12.04% and 69.23% for 100 ng/mL at 48 h and 72 h, and an increase of 12.04% and 139.74% for 1000 ng/mL at 48 h and 72 h, respectively, were observed, which indicate that the greater the concentration of leptin, the greater the increase in cell population. Likewise, the cells to which 4 stimuli of leptin were administered (96 h) showed a major decrease in cell population with regard to the control (10 ng/mL: 45.09%; 100 ng/mL: 56.86%; and 1000 ng/mL: 64.71%), possibly due to a saturation of the cell receptors by leptin.

In the MCF-7 breast cancer cells of group 2, the effect of leptin over cell proliferation was positive, increasing up to 28.57%, except for the concentration of 100 ng/mL, in which at 72 h of stimulation, the cell population decreased up to 26.68% (10 ng/mL, 48 h) and was maintained until 72 h at the concentration of 1000 ng/mL, where there was a 3.45% increase with regard to the control. This effect could be due to the elimination of the cellular adaptation period, since the cells were left to grow for another 24 h before the addition of exogenous agent (leptin) to the medium, allowing the cells to grow stronger, so as to not be damaged by the protein.

In the HCC1937 breast cancer cells of group 1, the effect of leptin over cell proliferation was negative at all concentrations evaluated (57.14%). As in the case of the MCF-7 breast cancer cells, it was observed that this antiproliferative effect is due to the sensibility of the cells while being recultured and the immediate administration of leptin; since despite a decrease in the cell population during the first stimulation with leptin (24 h), in the second, there was a small increase in the population with regard to the cells obtained at 24 h of stimulation: for the concentration of 10 ng/mL, an increase of 60%; for 100 ng/mL, an increase of 40%; and for 1000 ng/mL, an increase of 28.33%. However, this effect was not maintained with posterior stimulations.

The aforementioned results demonstrate that the period of cellular adaptation interferes with the effect of leptin over MCF-7 and HCC1937 proliferation, in which the period of cellular adaptation posterior to culture is between 2 and 3 days. In these cell lines, there was a positive effect over cell proliferation at all concentrations administered, when the first stimuli was administered 48 h after cell culture. Based on these results, it was decided that a cell stimulation be done with leptin 48 hours after cell culture.

An important find from the detection of leptin, adiponectin, AdipoR1, and AdipoR2 mRNA in breast cancer cells through endpoint PCR was that in HCC1937, a triple-negative cell line with a mutation in BRCA1 [44], these proteins and receptors are also found to be expressed, information that had not been reported in the literature. The aforementioned means that indistinctly of the molecular classification of breast cancer to which the cell lines belong, they can express leptin, adiponectin, and its receptors, allowing for the possibility of a future treatment for triple-positive breast cancer patients, patients with negative breast cancer, and patients with triple-negative breast cancer with mutations in BRCA1.

As for the expression assay, in MCF-7 breast cancer cells, the expression of mRNA of AdipoR2 (2.423 ± 2.35) was greater than the expression of AdipoR1 (1.679 ± 1.76). For the stimulations with leptin, it was found that the expression of AdipoR1 mRNA decreased with regard to the control in the cells that received 100 ng/mL and 1000 ng/mL of up to 3.81%, while the cells that received a stimulus of 10 ng/mL showed a 64.03% increase in expression. Surprisingly, the expression of AdipoR2 mRNA was dramatically increased at all concentrations of leptin: 10 ng/mL (13.75 times), 100 ng/mL (9.23 times), and 1000 ng/mL (11.39 times). Once again, the concentration at which there was a greater increase in the expression of the adiponectin receptor was at the “normal weight” concentration (10 ng/mL), which could indicate that in a condition of normal weight (10 ng/mL), at a high level of AdipoR1 and AdipoR2, the adiponectin could successfully carry out its antiproliferative activity of cancer cells and, in this manner, generate a form of protection for the patient.

The reduction in the expression of AdipoR1 in MCF-7 breast cancer cells corresponds with that proposed in the hypothesis of the present work, since in cells that received higher concentrations of leptin (100 ng/mL = excess weight-obesity; 1000 ng/mL), a decrease in the expression of mRNA of the gen was observed with regard to the control, while the cells that received stimulation with a concentration corresponding to normal weight showed an increase in the expression of both receptors. On the other hand, the increase in the expression of AdipoR2 mRNA and the decrease in the cell population at all concentrations at 96 h of stimulation could indicate that the effective receptor for the *in vivo* signaling of adiponectin in the breast cancer tissue is AdipoR2; this corresponds to that found in 2009 by Jardé et al. [27].

Despite this evidence, the notion of the effective receptor for adiponectin is uncertain, since it has been found that when siRNA was used against AdipoR1, the antiproliferative activity of adiponectin in T47D cells was annulated [30]. Likewise, studies have established a relationship between the presence of AdipoR1 and a better diagnosis in gastric and colon cancers [50, 51].

With regard to the expression of AdipoR1 and AdipoR2 mRNA in HCC1937 breast cancer cells, as in the case of MCF-7 cells, the expression of AdipoR2 mRNA (1.336 ± 0.905) was greater than the expression of AdipoR1 (1.181 ± 0.67). However, contrary to that found in MCF-7, there was a decrease in the expression of AdipoR1 mRNA of up to 86.26% (10 ng/mL), when stimulated with leptin, and up to 50.3% (100 ng/mL) for the expression of AdipoR2 mRNA. These results correspond to that found in cell viability curves (Figure 4), where it can be seen, in a much more marked manner, that there is a positive stimulation over cell proliferation at all concentrations of leptin employed due to the lack of antiproliferative activity offered by adiponectin through its receptors, generated from the decrease in the mRNA expression of these.

For HCC1937 cells, a part of the hypothesis proposed was corroborated for AdipoR1 and AdipoR2, due to the fact that there was a decrease in the expression of mRNA in both cell receptors in the cells that received stimulation from leptin every 24 h. Contrary to what is expected, a greater decrease of expression was found in concentrations corresponding to normal weight (10 ng/mL) and excess weight-obesity (100 ng/mL), which could indicate that in a condition of excess weight and obesity, there is a blockage in the expression of these receptors, which leads to adiponectin not being able to carry out its protective (antiproliferative) action during the development of breast cancer.

In our study, one of the limitations found was that the effect of leptin on cell proliferation and expression of AdipoR1 and AdipoR2 was modest, due to the range of leptin concentrations used to perform the cellular stimulus was very wide, which did not allow to identify exactly the effect that this protein has on the gene expression of these receptors in a state of obesity. On the other hand, it is impossible to extrapolate the results obtained from leptin stimulation in the cell lines with the results of expression that could be obtained directly from obese patients with breast cancer due to the complexity of the metabolic environment of the tumor *in vivo.*

## 6. Conclusion

In this study, the effect of leptin over cell proliferation and genic expression of AdipoR1 and AdipoR2 in MCF-7 and HCC1937 cells was modest. Indistinctly of the molecular classification of breast cancer of the cell lines used, they all express leptin, adiponectin, AdipoR1, and AdipoR2. It was determined that the period of cell adaptation generates an interference with the effect of leptin over the proliferation of MCF-7 and HCC1937 cells. During the development of breast cancer, the capacity of proliferation (23.68% for MCF-7 and 17.24% for HCC1937), explained by the deregulation of leptin in obesity, could be very relevant at the moment of prescribing a treatment to the patient. The concentration of leptin that generated an increase in the expression of both receptors of adiponectin was that corresponding to normal weight (10 ng/mL), indicating that in said condition, adiponectin can successfully carry out its antiproliferative activity over cancer cells and, in this manner, generate a form of protection for the patient.

The reduction in the expression of AdipoR1 mRNA and the increase in the expression of AdipoR2 mRNA could indicate that the effective receptor for the *in vivo* signaling of adiponectin in breast cancer tissue is AdipoR1.

## Figures and Tables

**Table 1:** Relative expression of AdipoR1 and AdipoR2.

Cell line	Leptin concentration (ng/mL)	2^−ΔΔCT^ AdipoR1	2^−ΔΔCT^ AdipoR2
MCF-7	10	2.754 ± 0.701	33.31 ± 16.006^∗∗^
	100	1.63 ± 1.74	22.354 ± 14.006^∗∗^
	1000	1.615 ± 1.209	27.607 ± 21.102^∗∗^
HCC1937	10	0.162 ± 0.129^∗∗^	1.01 ± 0.755
	100	1.019 ± 1.423	0.664 ± 0.502
	1000	0.27 ± 0.27^∗^	0.986 ± 0.597

2^−ΔΔCT^ Relative expression using BACT as a constitutive gene. The data is expressed as mean ± SD, *n* = 6. In statistical Mann–Whitney *U* test, significance with regard to the control ^∗^*p* ≤ 0.05, ^∗∗^*p* ≤ 0.01.
